# Understanding mental health difficulties and associated psychosocial outcomes in adolescents in the HIV clinic at Kenyatta National Hospital, Kenya

**DOI:** 10.1186/s12991-018-0200-8

**Published:** 2018-07-10

**Authors:** Douglas Gaitho, Manasi Kumar, Dalton Wamalwa, Grace Nduku Wambua, Ruth Nduati

**Affiliations:** 1 Department of Paediatrics, College of Health Sciences, University of Nairobi, P.O. Box 19676, Nairobi, Kenya; 2 Department of Psychiatry, College of Health Sciences, University of Nairobi, P.O. Box 19676, Nairobi, Kenya

**Keywords:** Adolescents, HIV, Psychosocial characteristics, Depression, School performance, Adherence

## Abstract

**Background:**

Globally adolescents continue to have an upward trend in HIV incidence and AIDS-related mortality. The interplay between the rapid physical growth, sexual maturation, and enormous albeit slow-evolving cognitive and psychological changes in adolescence may partly explain this trend. Our main purpose was to highlight key psychosocial characteristics of HIV-infected adolescents and explore if these characteristics are associated with depression symptoms.

**Methods:**

From August to December 2016 after obtaining informed consent, adolescents living with HIV at Kenyatta National Hospital were interviewed using the Home environment, Education and Employment, Activity, Sexuality, Suicide and depression traits (HEADSS) tool combined with the Patient Health Questionnaire (PHQ-9) to elucidate which key symptoms of depression and link with psychosocial characteristics mapped on HEADSS. In order to determine which psychosocial characteristics were linked with risk of depression, the traits of adolescents who were symptomatic were compared to those who were not using univariate and multivariate regression analysis.

**Results:**

All the 270 adolescents offered participation in the study accepted to enroll. The aged 10–19 years were recruited and mean age was 14.75 and 53.7% (*n* = 145) were males. Overall, 269 (99.9%) were still in school and 52.6% of the adolescents had symptoms of depression. The independent predictors of depression were being of ages 15–19 years [OR = 2.34 (95% CI 1.36, 4.04) *P* < 0.02], ever repeating classes [OR = 1.74 (95% CI 1.0–3.05) *P* = 0.05], ever being sent away from school due to lack of school fees [OR = 1.71 (95% CI 1.0–2.91) *P* = 0.05], and non-adherence to medication [OR = 1.84 (95% CI 1.08–3.14) *P* = 0.03. Missing of meals due to food insecurity was associated with an important trend towards increased risk of depression [OR = 2.42 (CI 0.96–6.14) *P* = 0.06].

**Conclusion:**

One in two of the adolescents interviewed had depression symptoms which were significantly associated with lack of school fees, missing meals, non-adherence to medication, and substance abuse.

## Background

Of the 2.1 million HIV-infected adolescents, 83% of these reside in sub-Saharan Africa [1]. In 2013, for the first time age-disaggregated estimated numbers of adolescents living with HIV (ALHIV) were presented in a global report that estimated 250,000 new HIV infections and 120,000 AIDS-related deaths among adolescents in that year [1]. Further to this, adolescents were the only age group in which AIDS-related deaths had not decreased in the period from 2005 to 2013 contrasting sharply with the reported cumulative 38% decline in all the other age groups [2]. An additional observation was the very low scale up and treatment of ALHIV.

The global *All In campaign to end adolescent AIDS* was officially launched in Kenya in February 2015 [2]. Kenya then released a *Fast Track Plan to End Adolescent AIDS* in September of the same year [3].

Implicit in the planned response is the assumption that adolescents will be able to take charge of their health care and all interventions would be multisectoral. Additionally, there is a tendency to lump all adolescents together failing to take into consideration that adolescence is a period of rapid growth and that there is significant lapse in time between sexual maturation taking place in adolescence (10–14 years) and/or emotional and cognitive maturation that takes place in late adolescence (17–24 years). This period of human development has well-described complex challenges that the HIV-infected adolescents have to get attuned to in addition to the problems related to their illness. Given that mental health issues are an emerging public health priority with suicide ranked as third highest cause of death among adolescents globally [4], ALHIV have higher prevalence rates of depression, anxiety, conduct, and functional disorders compared to HIV-unaffected adolescents [5–7]. HIV-related psychosocial problems include stigma and discrimination, relationship challenges such as HIV status disclosure, difficulties with their medication, loss of biological parents in cases of orphans, denial and ambivalence towards HIV, and school performance [8–10]. These have a major impact in the quality of life and if not adequately addressed can lead to poor outcomes [11]. These psychosocial adversities impact negatively on adherence to combined antiretroviral (cARV) medication [12, 13] and predispose adolescent greater depression risk. Risk factors such as being female, fewer years of schooling, death within the household, poor school performance, being in a relationship with the opposite sex, non-disclosure of HIV status, severe immunosuppression, and bullying in school for taking medication have been shown to be associated with depression [14].

Adolescence and especially mid-adolescence is characterized by increased exploration of one’s environments and feelings of invincibility. In the process, a significant number of adolescents engage in risky behaviors including high-risk sexual encounters that expose them to sexually transmitted infections and unplanned pregnancies [Ndugwa et al., Patel et al.]. Experimentation with psychoactive agents such as alcohol and others may lead to addiction and fuel poor sexual-decision making. ALHIV have the additional stress of putting their partner at risk, challenges with disclosure of HIV status, and adverse drug reactions from the psychoactive agents or with use of hormonal contraceptive [14, 15].

The purpose of this study was to highlight key psychosocial characteristics of HIV-infected adolescents and explore if these characteristics predict depression. The WHO AA-HA 2017 recommends competencies and intervention implementation targeting different developmental age groups and using HEADSS framework as a guidance tool. We feel our work aligns closely to the WHO adolescent care framework [4], as understanding and describing these psychosocial characteristics of HIV-infected adolescents will aid in design and implementation of treatment and follow-up programs that are tailor-made for their needs, thereby reversing the negative trends in this age group (notably these are reducing risky sexual behavior and consequently reducing new HIV infections, addressing issues pertaining disclosure, adherence to medication, and stigma/discrimination).

## Methods

### Study design

This was a cross-sectional descriptive survey among HIV-infected adolescents, attending a Comprehensive Care Clinic at Kenyatta National Hospital in Nairobi. The research team developed a semi-structured questionnaire based on the HEADSS framework. The tool screened information about the home environment, involvement in exercise and activity, school performance, use of drugs and substances of abuse, involvement in sexual activity, and depression/suicide. The screen for depression used questions obtained from the Patient Health Questionnaire (PHQ-9) which was administered to all adolescents. The HEADSS assessment tool has been validated in Outpatient Departments in the United States of America and found to accurately screen for mental health problems (MHP) with reported sensitivity of 82% and a specificity of 87% in predicting psychiatric consult and admission to in-patient psychiatry [16]. It has, however, not been validated in our setting. It is for this reason we did not use a cut-off.

### Study variables

The specific variables in the various domains of socio-demographic characteristics, Home environment, Education, Activity and routine, Drugs, Sexuality, Suicide and depression, Adherence to ARV drugs, and full disclosure of HIV status are shown in Table 1.

**Table 1:** Psychosocial characteristics evaluated using HEADSS framework

1. *Socio*-*demographic characteristics*—age, sex, education level, relation to the caregiver
2. *Home environment*—relationships with other household members, changing homes in the last 1 year, missing meals, and physical violence
3. *Education*—school performance, bullying, repeating classes, inability to pay school fees, change of schools in the last 2 years, and reasons for missing school
4. *Activity and routine*—involvement in extracurricular activities and religious activities
5. *Drugs*—use of drugs and substances of abuse
6. *Sex*—involvement in sexual activity, practice of safe sex, and worry about pregnancy/sexually transmitted diseases. Questions on sexual activity were restricted to adolescents aged 12 years and above
7. *Suicide and depression*—Any adolescent with a score of 1 or more on PHQ-9 was considered to have a depressive symptom
8. *Adherence to ARV drugs*—any positive response on any of the following 5 questions—do you ever refuse or miss drugs, don’t take drugs in front of others, or have problems taking drugs daily or on time—was taken to be an indication of non-adherence
9. *Full disclosure of HIV status*—an adolescent was classified as having full disclosure if they listed HIV in responding to any of the following 3 questions—why are you visiting the clinic, due to which illness, and why are you taking medication?

### Study population and setting

Adolescents were eligible for the study if they were aged 10–19 years. The study was carried out between August and December 2016. Ethical approval was obtained from The Ethics and Research Committee of Kenyatta National Hospital and University of Nairobi (approval no. P217/03/2016). All 270 participants who were approached consented to participate.

### Study procedure

Adolescents were offered study participation consecutively on a day they had attended a regularly scheduled clinic appointment. Written informed consent and assent was obtained from the caregiver and the adolescent, respectively. After this, the caregiver was interviewed first in the absence of the adolescent, and then the adolescent was interviewed on the expanded survey. The interview was carried out by the first and fourth authors according to the guidance of a senior psychiatrist consultant. The interview process lasted approximately half an hour. Those who experienced distress or had high PHQ-9 scores were referred to a psychiatrist immediately and received further psychosocial support at the clinic.

Study sample size was determined using Fischer’s Formula from previously published estimates of depression. Kim et al. [14] found the prevalence of depression among ALHIV to be 18.9%. By this token, our study was adequately powered to determine prevalence of depression.

### Data analysis

The data were collected on a paper tool and then entered onto SPSS. Descriptive statistics was computed for the study population, continuous variables, and frequency and proportions for categorical variables were determined. Univariate and multivariate linear regression analysis was carried out to determine factors associated with depression. In the univariate analysis study, participants were characterized as having depression versus no depression. Chi-square statistics were used to determine whether there was an association between depression and the various participant variables collected using the HEADSS framework. Variables that had a p value of < 0.05 in univariate analysis were entered into a multivariate linear regression to determine the independent predictors of depression. A Pearson’s correlation analysis was carried out to safe-guard against collinearity. In this analysis, depression was treated as a continuous variable so as to increase the power of the study to find associations. We have reported beta values, odds ratios, and the 95% confidence intervals.

## Results

Table 1 depicts the key characteristics of the study participants. The mean age of our sample of 270 was 14.75 years (SD = 2.6 years). This included 125 adolescents aged 10–14 years and 145 aged 15–19 years, of whom 145 (53.7%) were male and 125 (47.3%) female participants (Table 2).

**Table 2:** Factors associated with depression symptoms among HIV-infected adolescents on cARV and long-term follow-up at Kenyatta National Hospital

Parameter	Overall (*N* = 270)	Depression symptoms		OR (95% CI)	Chi square (*P* value)
		No (*n* = 128)	Yes (*n* = 142)		
Gender					
Male	145 (53.7)	70 (54.7)	77 (54.2)	Ref.	(*χ*^2^_(1, 270)_ = 0.01; *P *= 0.939)
Female	125 (46.3)	58 (45.3)	65 (45.8)	1.0 (0.6–1.7)	
Age					
10–14	125 (46.3)	75 (58.6)	50 (35.2)	Ref.	(*χ*^2^_(1, 270)_ = 14.80; *P *= 0.001)
15–19	145 (53.7)	53 (41.4)	92 (64.8)	2.60 (1.6–4.3)	
School environment					
Happy with school performance	193 (71.5)	92 (71.9)	101 (71.1)	0.96 (0.6–1.6)	(*χ*^2^_(1, 270)_ = 0.02; *P* = 0.9)
Plan to continue with education	260 (96.3)	122 (95.3)	138 (97.2)	1.70 (0.5–6.2)	(*χ*^2^_(1, 270)_ = 0.66; *P* = 0.4)
Been bullied in school	50 (18.5)	21 (16.4)	29 (20.4)	1.31 (0.7–2.4)	(*χ*^2^_(1, 270)_ = 0.72; *P* = 0.4)
School performance dropping	49 (18.1)	20 (15.6)	29 (20.4)	1.39 (0.7–2.6)	(*χ*^2^_(1, 270)_ = 1.04; *P* = 0.3)
Changed schools in the past 2 years	85 (31.5)	33 (25.8)	52 (36.6)	1.66 (1.0–2.8)	(*χ*^2^_(1, 270)_ = 3.67; *P* = 0.05)
Ever repeated a class	91 (33.7)	34 (26.6)	57 (40.1)	1.85 (1.1–3.1)	(*χ*^2^_(1, 270)_ = 5.56; *P* = 0.02)
Ever sent away from school due to lack of fees	119 (44.1)	45 (37.8)	74 (62.2)	2.01 (1.2–3.3)	(*χ*^2^_(1, 270)_ = 7.85; *P* = 0.005)
Activity					
Has extracurricular activities	190 (70.4)	90 (70.3)	100 (70.4)	1.0 (0.6–1.7)	(*χ*^2^_(1, 270)_ = 0.00; *P* = 0.9)
Attend religious activities	251 (93.0)	120 (93.8)	131 (92.3)	1.26 (0.5–3.2)	(*χ*^2^_(1, 270)_ = 0.23; *P* = 0.6)
Home environment					
Changed homes in the last 1 year	59 (21.9)	28 (21.9)	31 (21.8)	1.00 (0.6–1.8)	(*χ*^2^_(1, 270)_ = 0.00; *P* = 0.99)
Ever ran away from home	18 (6.7)	4 (3.1)	14 (9.9)	3.39 (1.1–10.6)	(*χ*^2^_(1, 270)_ = 4.91; P = 0.03)
Physical violence at home	11 (4.1)	5 (3.9)	6 (4.2)	1.09 (0.3–3.7)	(*χ*^2^_(1, 270)_ = 0.02; *P* = 0.895)
Involuntarily misses meals because of lack of food	34 (12.6)	9 (7.0)	25 (17.6)	2.83 (1.3–6.3)	(*χ*^2^_(1, 270)_ = 6.84; *P* = 0.009)
Drug and substance use					
Family members or friends who use drugs	96 (35.6)	41 (32.0)	55 (38.7)	0.75 (0.5–1.2)	(*χ*^2^_(1, 270)_ = 1.32; *P* = 0.3)
Use drugs/substance abuse	23 (8.5)	5 (3.9)	18 (12.7)	3.57 (1.3–10.0)	(*χ*^2^_(1, 270)_ = 6.64; *P* = 0.01)
Sexuality					
Agree that condoms reduces HIV transmission	126 (46.7)	52 (40.6)	74 (52.1)	1.59 (1.0–2.6)	(*χ*^2^_(2, 270)_ = 3.83; *P* = 0.1)
Ever had sex (*N* = 228)	31 (13.6)	12 (12.4)	19 (14.5)	1.20 (0.6–2.6)	(*χ*^2^_(1, 228)_ = 0.22; *P* = 0.6)
Inconsistently using condoms (*N* = 29)	20 (69.0)	8 (66.7)	12 (70.6)	1.20 (0.2–5.9)	(*χ*^2^_(1, 29)_ = 0.05; *P* = 0.8)
HIV Disclosure					
Full HIV disclosure	174 (64.4)	78 (60.9)	96 (67.6)	1.34 (0.8–2.2)	(*χ*^2^_(1, 270)_ = 1.31; *P* = 0.3)
Adherence to medication					
Non-perfect adherence to medication	139 (51.5)	50 (39.1)	89 (62.7)	2.62 (1.6–4.3)	(*χ*^2^_(1, 270)_ = 15.02; *P* < 0.001)
Perfect adherence to mediation	131 (48.5)	78 (60.9)	53 (37.3)	Ref.	

Dividing key findings on the HEADSS framework, the following results are notable.

- *Home environment* Eleven (4.1%) study participants reported that they had encountered physical violence in their home, 18 (6.7%) reported running away from home at least once, and another 59 (21.9%) adolescents reported that they had changed homes in the past 1 year. In the past 2 weeks preceding the study, 80 (29.6%) reported they had involuntarily missed a meal.
- *Education* Overall 269 (99.6%) of the study participants were in school, 137 participants (50.7%) were enrolled in primary school, 98 (36.3%) in secondary school, and 35 (13%) participants in tertiary or vocational training institutions. Overall, 260 (96.3%) of the adolescents were committed to furthering their education. The commonest reason for ever missing school was sickness as reported by 265 (98.1%) of our participants. Bullying in school was reported by 50 (18.5%) study participants. A total of 91 (33.7%) study participants had repeated a grade, 77 (29%) participants reported dissatisfaction with their current school performance, and 49 (18.1%) participants had experienced a drop in their grades. A total of 119 (44.1%) participants had at least once been sent away from school due to lack of tuition fees.
- *Activity and routine* Up to 190 (70.4%) of the study participants were involved in extracurricular activities which included sports and other hobbies. Almost all of the study participants, 251 (93%), attended religious activities.
- *Drugs* Only 23 (8.5%) acknowledging use of drugs and substances of addiction, while 96 (35.6%) reported use of the same among family or friends.
- *Sexual activity* There were 229 study participants aged 12 years and above who were interviewed regarding their sexual activity. Among this group 31 (13.6%) reported they had achieved sexual debut, including 10 (35.7%) participants for whom first sexual encounter was at 18 years of age. Overall, 19 (73%) participants of the sexually active adolescents had one sexual partner. Of those who reported had ever had sex, 20 (64.5%) participants reported inconsistent condom use. Sexually active adolescents obtained condoms from health facilities. Eight (3%) adolescents reported having a forced sexual encounter. This means 8 of the 31 (26%) sexual debuts may have been through a forced sexual encounter or abuse of some kind.
- *Suicide and depression* Overall, 142 (52.6%) had at least one symptom of depression. The mean depression score was 1.78 (SD = 2.81). Twelve (4.4%) of the adolescents were found to have suicidal ideation in the last 2 weeks preceding the survey. Suicidal adolescents were immediately referred to a psychiatrist, while the others were referred to a specialist counselor based within CCC and all these cases received psychiatric oversight.
- *Drug adherence* Overall, 139 (51.5%) of the study participants reported a non-perfect adherence to their medications.

### Univariate analyses of risk factors for depression

Male and female adolescents had similar levels of depression symptoms. Adolescents aged 15–19 years had a significantly higher prevalence of depression symptoms compared to those aged 10–14 years, 92 (63.4%) versus 50 (40%) of our total sample [OR = 2.6 (CI 1.6–4.3)*, P *< 0.001)]. Depression was significantly associated with frequent changing of schools in the preceding 2 years due to repeated adversities with 52 (61.2%) of 142 versus 33 (38.8%) of 128 participants, [OR = 1.66 (95% CI 0.99–2.81)*, P* = 0.05]. Depressed adolescents were significantly more likely to report ever repeating a grade as compared to those who were not depressed with 57 (62.6%) of 142 versus 34 (37.4%) of 128 [OR = 1.85 (95% CI 1.11–3.11)*, P* = 0.02*].* A significantly higher proportion of adolescents with symptoms of depression reported that they had ever been sent back home for lack of school fees 74 (62.2%) of 148 compared to 45 (37.8%) of 128, who had no symptoms [OR = 2.01 (95% CI 1.23–6.31)*, P* = 0.005].

In the home environment, 25 (18.3%) of 142 adolescents experiencing symptoms of depression reported that they involuntarily missed meals due to unavailability of food compared to 9 (7%) of 128 without such symptoms, [OR = 2.83 (95% CI 1.27–6.31), *P* = 0.009]. Adolescents who were depressed were more likely to report that they had at least once ran away from home 14 (9.9%) versus 4 (3.1%), [OR = 3.39 (95% CI 1.09–10.58)*, P* = 0.03*].* Adolescents who were depressed had a significantly higher likelihood of reporting substance use compared to those who had no symptoms of depression 18 (12.7%) versus 5 (3.9%), [OR = 3.57 (95% CI 1.29–9.92), *P* = 0.01]. Use of drugs by family members was not associated with symptoms of depression. Adolescents who had experienced any symptom of depression were significantly more likely to report non-perfect adherence to their medications 89 (62.7%) versus 50 (39.1%), [OR = 2.62 (95% CI 1.60, 4.28)*, P* ≤ 0.001].

### Multivariate analysis of association between PHQ-9 scores and other variables

The independent correlates of depression in the group of adolescents were being of ages 15–19 years [OR = 2.34 (95% CI 1.36–4.04), *P* < 0.02], having had an experience of repeating a grade [OR = 1.74 (95% CI 1.0–3.05) *P* = 0.05], having had an experience of being refused school participation due to lack of school fees [OR = 1.71 (95% CI 1.0–2.91) *P* = 0.05], and non-adherence to medication [OR = 1.84 (95% CI 1.08–3.14) *P* = 0.03] (Table 3).

**Table 3:** Correlates of depression symptoms

Parameter	β	S.E	A.O.R	95% CI		*P* value
				Lower	Upper	
Age in years 15–19 years	0.85	0.28	2.34	1.4	4.0	0.02
Changed schools in the past 2 years	0.39	0.29	1.47	0.8	2.6	0.2
Ever repeated a class	0.56	0.28	1.74	1.0	3.0	0.05
Ever sent away from school due to lack of fees	0.54	0.27	1.71	1.0	2.9	0.05
Ever ran away from home	0.34	0.65	1.40	0.4	5.0	0.6
Involuntarily misses meals because of lack of food	0.89	0.47	2.42	1.0	6.1	0.06
Use drugs/substance abuse	0.55	0.58	1.72	0.6	5.4	0.3
Non-adherence to medication	0.61	0.27	1.84	1.0	3.1	0.03

## Discussion

This study is among the few in Kenya to comprehensively assess at the health facility various psychosocial issues that are faced by ALHIV. Of importance is that these issues have not become part of routine assessment and management during regular clinic visits and these psychosocial correlates in the literature have been strongly associated with depression. The HEADSS tool framework was adopted into the adolescent tool kit called Adolescent Package of Care (APOC) by the Kenya Ministry of health to support care of adolescents; however, the specific questionnaire has not been adapted into the Kenyan context yet [17]. The standard HEADSS tool was shortened and adapted to the Kenyan context and a standard depression screening tool added. The tool is included as supplementary study material. This is the first study to provide empirical evidence on the performance of this HEADSS framework in assessing ALHIV since this framework was adopted in APOC guidelines. As noted in our introduction, WHO AA-HA (2017) stipulates the use of HEADSS tool for not only needs assessment but as a framework for intervention development looking at different developmental competencies and domains in which adolescent development and health care needs have to be conceptualized [4].

In line with this, our work despite its shortcoming and narrow scope (being restricted to health facility) has high operational validity as we have directly explored with adolescents about their ongoing treatment and mental health needs. We have bifurcated analyses based on differential developmental competencies [18]. National adolescent priorities have been laid out in a number of complementary guidelines. The Kenyan national adolescent reproductive and sexual health policy [18], Vision 2030 [19], and UN SDGs such as goals focus on adolescent health. The Kenyan national policy focuses on adolescents’ needs that have been specifically targeted such as reducing early sexual debut, knowledge about HIV transmission, and prevention in adolescent girls and boys and in adolescents of ages 10–14 and 15–24 years.

We evaluated 270 adolescents and a wide majority (99.6%) as would be expected were still in formal education at different tiers. A study done by Souza et al. (2010) had a higher dropout rate, with 89.8% in school in the same population in Brazil [10]. Despite high numbers of those in formal education, the challenges came in the form of financial constraints that came in the way of parental capacity to pay for school fees in time. In our sample, 44.1% alluded to missing school due to unpaid school fees which prompted the school to discontinue these candidates. Additionally, there were also concerns about dropping school performance in 18.1% of our sample with 33.7% having repeated a grade and 18.5% having had difficult bullying experiences at school. In a study by Shavisa et al. [20], school dropout rates in the general population were 1.34 and 1.06% among boys and girls, respectively. The shame associated with poor performance and failure in tests may have exacerbated experiences of bullying. Bullying of vulnerable child who live in adversities also needs greater attention. A systematic review done found that problems with school functioning need early detection and intervention and prolonged problems may lead to long-term adjustment and psychological disorders [10].

Our study found older adolescents (age 15–19 years) with a twofold increased risk of depression. Adolescence is a period of significant cognitive and socio-emotional development [21]. Mental illness may manifest in a different way as these capacities develop. Given the complex pathogenesis of HIV illness, mental health problems, poor neurocognitive functioning, and psychosocial adversities in such vulnerable adolescents are subsets of an inter-related problem. Additionally, due to the HIV infection, these developing capacities are also compromised in ways that are also yet to be fully understood in both neurocognitive and psychosocial senses. Our cross-sectional study design does not equip us sufficiently to extend this argument based on these results but the literature is replete with similar conclusions [12]. In this study, we used PHQ-9 to screen for depression symptoms and as such we did not find depression prevalence to be high, although we did find that 12 (4.4%) participants reported suicidal ideation whom we referred for suicide watch and priority mental health care. The use of a standardized check list to screen for symptoms of depression was therefore a huge benefit for this set of study participants.

The other independent predictors for depression were repeating a class and poverty measured by the surrogate markers that included missing meals and being sent away from school due to inability to source school fees. There is emerging evidence from the National HIV program that school environment frequently destabilizes the adolescent leading to poor drug adherence [22]. In this study, we show two school-related factors that were associated, repeated changing of schools and being sent away due to lack of school fees, both factors connected to the material conditions of the families.

One in two of the adolescents in the study were not adherent to their medication. The measure of adherence in this study was a crude one in that it relied on self-reporting. A study by Vreeman et al. [23] comparing the use of Medication Events Monitoring System (MEMS) versus self-reported adherence demonstrated over-reporting in the latter group. Further to this, non-adherence was one of the independent predictors of depression symptoms. In this cross-sectional study, we cannot reliably say what came first. There may be merit in offering psychotherapy immediately to ALHIV who have symptoms of depression as a preventative measure.

One in three adolescents live in an environment where they are exposed to drugs and other substances of abuse. One in ten of the adolescents interviewed reported substance abuse with alcohol being the most commonly used. The use of drugs increased the presence of depression symptoms by 70%. These findings are very critical as related studies have found a strong association between mental health problems, risky sexual behaviors, and substance abuse [24] in both HIV- and non-HIV-infected adolescents. There should then be a deliberate effort to inform and counsel adolescents on the harms of drug and substance use. There is a need for further work on the tool to clarify whether the ALHIV really understand when we use the term ‘drugs’ in this context given the fact that they are on medication all the time.

The study found that involuntary missing of meals due to unavailability of food in the household was also associated with twofold increased risk of depression. People living with HIV are instructed to ensure that they do not miss meals as they take their cARV and we commonly find that people attribute non-adherence to paucity of food and to a fallacy that given the food insecurity, taking drugs without food would be harmful for the body. All these experiences probably increase the sense of vulnerability and stigma for the adolescent who lives among so many social, financial, and emotional adversities. This study did not collect formative data that would better explain the impact of these factors on the adolescents and we do feel this is an important area to invest in. A study carried out in Malawi by Kim et al. [14] found bullying because of being on medication, severe immunosuppression, and having a boyfriend/girlfriend was associated with depression. This study did not find any significant associations between depression symptoms and bullying and we did not collect data on the state of immunosuppression.

Another important finding was that just under two-thirds of the adolescents have been fully disclosed of their HIV status. Surprisingly depression was not associated with disclosure in our univariate analysis (OR 1.34; *P* = 0.3). There is a need for further studies to validate this observation. Health care workers may be deferring disclosure to ALHIV to those who are overtly depressed to manage their mental health first before a proper disclosure is planned. Disclosure we know is also a process which needs pre-disclosure, disclose, and post-disclosure stage planning [25]. One of the key steps for ALHIV is to gradually take on responsibility for one’s own care. Full disclosure would then give meaning to the health education and actions that the adolescent is instructed to follow. The important questions before us are as follows: does disclosure trigger or worsen depression, and further to this does it have an impact on drug adherence if the disclosure experiences have been negative or ambivalent?

Although not associated with depression, just over one in 10 adolescents had made a sexual debut. Among those adolescents interviewed who were 12 years and older, 13.6% reported being sexually active or having had a sexual encounter. These experiences were more common among ages 15–19 years. According to Kenya Demographic and Health Survey 2014, 37.3% of adolescents aged 15–19 years reported ever having sex [26] a higher prevalence compared to our study participants suggesting that HIV-infected adolescents may be postponing sexual debut or have delays in development due to recurring or chronic illness. There is considerable variability in proportion of HIV-infected adolescents who have achieved sexual debut with a Ugandan study reporting a third [15], while in South Africa, Toska et al. [27] found a prevalence of 14.9% in their study sample. These differences may reflect variability in the age composition of the study population and also the prevalent norms on adolescent sexuality in different cultural settings.

In our study, the majority of the adolescents who had achieved sexual debut reported inconsistent condom use similar to a Ugandan study that showed 76.5% of ALHIV inconsistently used condoms [9], a finding that underscores the need to bolster sexual and reproductive health education in this group. Inconsistent use of condoms poses important challenges including reinfection of a HIV-infected partner with risk of transmitting resistant strains, transmission of HIV to their uninfected partners, and acquisition and transmission of sexually transmitted diseases. In our study, 27% of adolescents did not think that condoms reduced HIV transmission, while another 26.3% did not know if condoms reduced transmission of HIV. This is also an alarming response from our participants whose knowledge about sexual protection appeared to be deficient warranting a better framing of SRH education policy in schools and communities. The majority of adolescents in our study obtained condoms from health facilities; however, health care workers did not feature as a key source of information about HIV and other reproductive health issues. This maybe an indication of limited contact between adolescents and health workers or missed opportunities within the health facilities or lack of a structured method for collecting information like this study did using the HEADSS framework.

We think that the HEADSS framework offered us an opportunity to identify the adolescents who needed more immediate or focused services and further management. An alarming finding in this context was that one in four sexual debuts might have been due to a forced encounter or due to abuse. This clearly conveys the evidence of adverse childhood experiences that need to be understood better and adolescents who have had an adverse experience of coerced sex or abuse need emergency services, counseling, and socio-legal protection from further abuse. The adolescent clinic can be the point of referral for such young persons to child protection services who would need to be more responsive to needs of those young people who present with chronic illnesses or infections like HIV. Another notable finding is that about nine out of ten ALHIV are not sexually active and this offers a great opportunity to introduce psychoeducation and preventive health services that such adolescents could benefit from. Building their parents/guardians’ or health worker skills on how to support their adolescent to adopt safe approach to their sexuality would be the next step.

This study has several strengths that include a validated adolescent evaluation framework such as HEADSS. The study was conducted in a well-established clinic that had followed many of the adolescents since childhood in the context of their HIV care. All the adolescents were on antiretroviral drugs. The study was conducted during the school holidays so that adolescents in boarding school had an equal opportunity to participate similar to those in day schools. All the adolescents approached for the study accepted to participate and so did their guardians indicating that there is minimal non-participation bias and eagerness to share experiences in this group. In order to ensure informed consent and respect for the relationship between the adolescent and the parent/guardian, the researchers first approached the guardian for permission before assent by the adolescent. This overwhelming participation probably reflects that young people are very responsive to mental health care services and do have a strong desire to engage with providers on addressing their needs.

In published literature, the PHQ-9 tool has been validated among non-HIV-infected adolescents in the USA [16]. In Africa, this tool has been used among HIV-infected adults in Western Kenya [20] and among adolescents living with HIV in Tanzania. In the latter study, severity of depression was computed as score of 10 or greater based on the validation studies done in adults in Western Kenya [12]. We are therefore reasonably confident that our estimates of depression in this study are valid. The adapted HEADSS tool enabled the identification of 12 adolescents with suicidal ideation. This was 4% of the study population and was the first time this diagnosis was made in these adolescents. This finding alone is a strong justification for routine use of HEADSS tool in assessing HIV-infected adolescents.

There is a need to delve further and make a DSM-V diagnosis of depression as part of the strategy to develop more refined criteria for diagnosis of major depression among ALHIV. This study contributes to the growing body of studies using standardized tools. There is a need for validation of this abbreviated tool against the comprehensive HEADSS and other measures of depression such as Beck Depression Inventory version II (BDI-II) and The Children’s Depression Rating Scale-Revised (CDRS-R).

A major limitation of the study was its cross-sectional design, meaning that exposure and outcome were measured at the same time, and therefore causal relationships could not be ascertained. Further to this, the study did not use the full-length HEADSS tool and therefore there is a need for further work to adapt the tool to our environment. Many of the questions posed in this tool are sensitive and there may have been some bias if adolescents engaged in face-to-face interviews under reporting or gave socially acceptable responses. Despite these limitations, the interviews tapped into important areas which need to be investigated in further details. The study did not have a comparison group of adolescents with a different chronic illness and therefore cannot establish whether the depression symptoms are unique to HIV-infected adolescents or a feature of adolescents struggling with chronic illness.

## Conclusion

Overall, our work highlights the need to tap into depression, school performance, adherence and engagement-related barriers and challenges, as well as sexual and reproductive health awareness needs. One in two ALHIV in our study had depression symptoms. HEADSS and PHQ-9 tools should be incorporated into routine care of adolescents. Further studies need to be conducted to determine causal relationship of the factors identified as the predictors of depression.

## Abbreviations

ALHIV: adolescents living with HIV
HEADSS: home environment, education and employment, activity, sexuality, suicide and depression traits
PHQ-9: Patient Health Questionnaire
MHP: mental health problem
SRH: sexual and reproductive health
cARV: combined antiretroviral
APOC: adolescent package of care
